# Superdense teleportation using hyperentangled photons

**DOI:** 10.1038/ncomms8185

**Published:** 2015-05-28

**Authors:** Trent M. Graham, Herbert J. Bernstein, Tzu-Chieh Wei, Marius Junge, Paul G Kwiat

**Affiliations:** 1 Department of Physics, University of Illinois at Urbana-Champaign, 1110 West Green Street, Urbana, Illinois 61801, USA; 2 Institute for Science & Interdisciplinary Studies & School of Natural Sciences, Hampshire College, Amherst, Massachusetts 01002, USA; 3 C.N. Yang Institute for Theoretical Physics and Department of Physics and Astronomy, State University of New York at Stony Brook, Stony Brook, New York 11794, USA; 4 Department of Mathematics, University of Illinois at Urbana-Champaign, 1409 West Green Street, Urbana, Illinois 61801, USA

## Abstract

Transmitting quantum information between two remote parties is a requirement for many quantum applications; however, direct transmission of states is often impossible because of noise and loss in the communication channel. Entanglement-enhanced state communication can be used to avoid this issue, but current techniques require extensive experimental resources to transmit large quantum states deterministically. To reduce these resource requirements, we use photon pairs hyperentangled in polarization and orbital angular momentum to implement superdense teleportation, which can communicate a specific class of single-photon ququarts. We achieve an average fidelity of 87.0(1)%, almost twice the classical limit of 44% with reduced experimental resources than traditional techniques. We conclude by discussing the information content of this constrained set of states and demonstrate that this set has an exponentially larger state space volume than the lower-dimensional general states with the same number of state parameters.

## Abstract (web summary)

Implementations of known quantum teleportation techniques suffer from a number of technical limitations, most notably the scaling of the required classical resources. Here, the authors implement a new protocol, superdense teleportation, which requires fewer resources than the conventional approaches.

## Graphical abstract

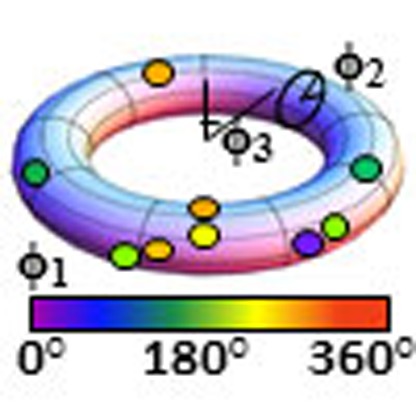

The transfer of quantum information over long distances has long been a goal of quantum information science. Loss is particularly devastating to quantum communication channels as quantum states cannot be amplified^1^. Moreover, random fluctuations in the communication channel can reduce the coherence of a quantum state, and error correction protocols for quantum states are presently very difficult to implement in practice^2^. However, if the sender (Alice) and the receiver (Bob) already share an entangled pair of qubits, then they may use a number of techniques to transfer quantum states using only classical information channels. In single-qubit quantum teleportation (QT, Fig. 1a)^3^, Alice performs a measurement in the Bell state (that is, maximally entangled) basis on the unknown state provided by a state chooser (Charles) and her half of the entangled state that she shares with Bob. She then sends the two-bit outcome of her measurement to Bob over a classical communication channel. On the basis of Alice's message, Bob performs one of four unitary transformations on his half of the originally entangled pair, transforming it into the exact state that Charles chose. Teleportation has been successfully demonstrated with probabilistic protocols for photons^4^,^5^,^6^,^7^ and with deterministic protocols using nonlinear interactions for ions, atoms, superconducting qubits and hybrid systems between photons and ions^8^,^9^,^10^,^11^,^12^. More recently, QT has been performed using photons entangled in spatial mode where Charles' quantum state is encoded on the polarization degree of freedom of Alice's photon^13^. Since Bell measurements between photonic degrees of freedom (of the same photon) do not require nonlinear interactions, this protocol could theoretically be implemented with 100% efficiency.

In teleportation, Charles provides a quantum state that he wishes to be sent to Bob. However, if Charles is instead allowed to encode his desired state parameters he wishes to send directly on Alice's half of the entangled state, then a simpler method may be used to transmit the unknown qubit state from Alice to Bob. In this technique, known as remote state preparation (RSP, Fig. 1b), Alice needs only to perform measurements on a single qubit and transmit the outcome to Bob^14^. Then, as in teleportation, Bob performs a unitary transformation on his qubit, based on the message he received. It might be speculated, since Alice performs her measurement only on a single-qubit state, that she would only have to send a single-bit message to Bob. However, because Bob cannot perform a universal NOT gate (a mapping of the input state to its orthogonal)^15^, a one-bit message from Alice is generally not sufficient for him to convert his state to the one Charles wished to send^14^. In fact, because of the impossibility of a universal NOT gate for general qubits, most RSP implementations are inherently probabilistic^16^,^17^; moreover, as the dimension of the remotely prepared state increases, the probability of success becomes smaller. To remotely prepare quantum states deterministically, Alice must instead perform a positive-operator valued measure (POVM) measurement on her quantum state and send the outcome message to Bob^18^. With this larger message from Alice (2 bits for qubit RSP), Bob can transform his state into the state Charles chose using simple unitary operations. Deterministic RSP protocols have been implemented for photon and ion qubit states^19^,^20^,^21^.

While both QT and RSP allow Alice to communicate quantum information to Bob using shared entanglement and a two-bit classical message, each technique has advantages and disadvantages. Because QT requires a full Bell-state measurement, it is impossible to implement deterministically in linear optical systems^22^,^23^; in contrast, RSP only requires Alice to make measurements using linear optics (which can be made deterministically). On the other hand, QT does not require even Charles to know what state he is sending to Alice, enabling him to implement entanglement swapping^24^, which cannot be accomplished using RSP. For both higher-dimensional QT and RSP, the classical communication cost can be shown to scale as log_2_ *d*^2^ with the dimension *d* of the quantum state that has 2*d*–2 continuous state-defining parameters (such as, *θ* and *φ* in cos *θ*|0〉+*e*^*iφ*^ sin *θ*|1〉) (ref. 14).

Here, we report an implementation of a new quantum communication protocol, known as superdense teleportation (SDT), which has reduced classical information resource requirements compared with QT, simplified measurements for Alice, easier transformations for Bob and can in principle be implemented deterministically in (linear) optical implementations^25^. We first describe the SDT protocol and compare its resource requirements with QT and RSP. Next, we report how we use spontaneous parametric downconversion to create photon pairs hyperentangled in polarization and orbital angular momentum. We then outline our experimental procedure for implementing SDT using hyperentangled photons, confirming that quantum information was transmitted using two-qubit single-photon tomography. Finally, we discuss the information content of the specific class of inputs states used in SDT and compare with general states with the same number of state parameters.

## Results

### Superdense teleportation resource analysis

The two state-transfer techniques described in the previous section are used to send completely general quantum states. However, it is possible to remotely prepare qubit states that are constrained to lie on a great circle of the Poincaré sphere, requiring only a single bit transferred from Alice to Bob^26^,^27^,^28^. Furthermore, this idea of transmitting a state from a constrained portion of Hilbert space may be extended to higher-dimensional states^29^,^30^; the resulting technique, SDT, can be used to send states at a reduced classical information cost per state parameter^25^. SDT is somewhat similar to the standard RSP protocol, in that Charles encodes the state parameters that he wishes to communicate to Bob directly onto Alice's half of the entangled state. However, unlike traditional RSP, instead of attempting to send a general *d*-dimensional state, requiring all 2*d*–2 state-defining parameters, Charles only attempts to send a state with *d*–1 state-defining parameters, corresponding to the relative phases of an equimodular state (also known as an equatorial qudit):


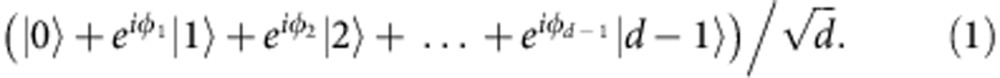


To do this, he applies these phases to the input maximally entangled state, resulting in,


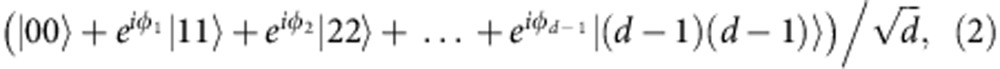


and sends his modified half of the entangled state to Alice. She then measures her qudit (*d*-dimensional quantum state) in a basis that is mutually unbiased to the one Charles used to apply the phases, and sends the measurement outcome, only log_2_ *d* bits, to Bob, who performs one of *d* relative-phase-shifting unitary transformations on his particle to recover the intended state (1).

The reduction in classical information required by SDT is not only interesting from a theoretical point of view but is also accompanied by significant experimental simplifications. Chief among these is the reduced complexity of the measurements (for example, number of interferometers and detectors) that Alice must make on her half of the entangled state. The measurements required for QT are probabilistic when using linear optics; this problem is worsened when teleporting higher-dimensional states. Because the percentage of the total higher-dimensional Bell states discriminated with linear optics detection decreases as dimension increases, QT of states *d*>2 is impossible to do with perfect fidelity without nonlinear interactions^31^,^32^. Furthermore, although RSP can be performed deterministically for any state dimension, the complexity of the measurement increases quadratically with the state dimension: a *d*-dimensional state with 2*d*–2 state parameters requires a POVM with *d*^2^ outputs and detectors. SDT, in contrast, requires only a comparatively simple *d*-dimensional mutually unbiased basis measurement to teleport a *d*-dimensional state with *d*–1 state parameters. While SDT sends only half the number of state parameters associated with a *d*-dimensional state, the complexity of the experiment is greatly reduced. For example, the number of detectors scales linearly with the state dimension for SDT instead of quadratically as in RSP. Moreover, the number of different transformations Bob needs to implement is thus also reduced to linear scaling with the dimension—much easier than the quadratic scaling for RSP (and QT). Table 1 summarizes the three protocols.

### Hyperentangled state creation

To experimentally demonstrate SDT's advantages over QT and RSP (for example, reduced classical communication cost and experimental measurement simplification), states with at least two quantum parameters must be transferred^25^. Here we experimentally demonstrate SDT by transmitting equimodular ququart states (four-dimensional quantum states with three independent state parameters). This may be accomplished by preparing entangled states in four modes of one degree of freedom, such as a spatial or temporal mode. Instead, however, we use states that are hyperentangled—simultaneously entangled in multiple degrees of freedom—in polarization and orbital angular momentum to produce four-mode entangled states^33^.

**Figure 2:**
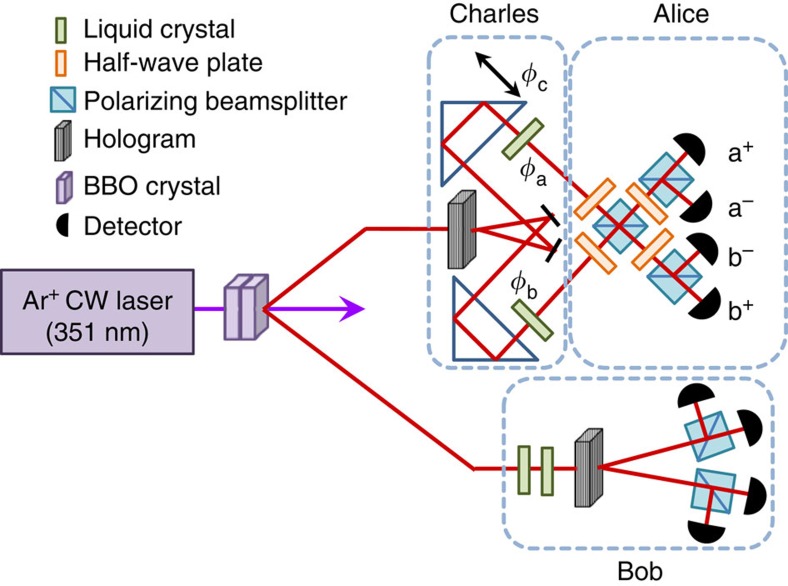
Experimental set-up for the SDT implementation. Charles applies phases to Alice's photon using liquid crystals and adjusting an interferometer path length. These phases are linear combinations of the phases given in equation (1) 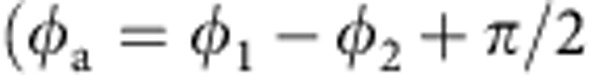, 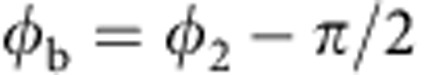 and 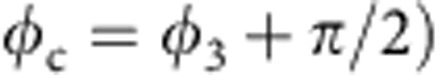, but still span the space of equimodular states that Charles can prepare. Alice then makes a single-photon two-qubit Bell-state measurement on the polarization and spatial mode of her photon^37^. By measuring in coincidence with Alice, Bob can determine the state heralded by each of Alice's measurements.

To create the required hyperentangled states, we pump a pair of nonlinear type-I phase-matched beta barium borate (BBO) crystals with a 351-nm Ar^+^ laser (see Fig. 2 and Methods for details). With rare probability, a high-energy photon may be split by the nonlinear crystals into two lower-energy photons through spontaneous parametric downconversion. These crystals were oriented such that a horizontally (vertically) polarized pump photon split in the first (second) crystal will produce two vertically (horizontally) polarized photons. By pumping the crystals with a coherent, equal superposition of horizontal and vertical polarization, we created a maximally entangled polarization state^34^. Furthermore, because orbital angular momentum is conserved in the downconversion process, the daughter photons will be correlated in orbital angular momentum as well^35^. By selecting only the ±*ħ* orbital angular momentum modes, we create a state that is maximally entangled in both polarization and spatial mode:


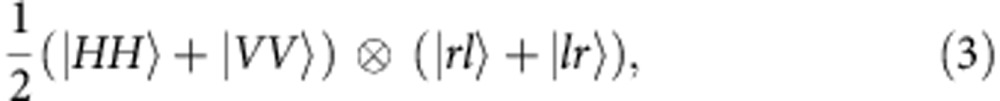


where *r* and *l* are eigenfunctions of the orbital angular momentum operator with ±*ħ* orbital angular momentum^33^. One photon of the resulting state was sent to Charles, and the other to Bob.

### Experimental implementation

To encode the three state parameters that Alice must teleport to Bob, Charles applied phases using liquid crystals and by varying the phase between the two spatial modes, which were processed using a binary forked hologram. These silver-halide holograms were used in conjunction with single-mode fibres to transform the *r* and *l* states into two Gaussian modes in the ±1 diffraction orders, respectively (with ∼30% efficiency)^36^. After these transformations, the total two-photon entangled state was:


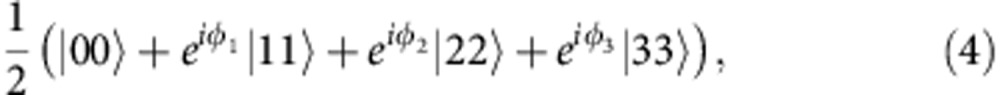


where |0〉≡|*Hr*〉, |1〉≡|*Hl*〉, |2〉≡|*Vr*〉, and |3〉≡|*Vl*〉 (in reality the labels *r* and *l* are reversed for Alice and Bob, but this does not affect the results). Charles then sent the photon to Alice, who combined the two spatial modes on a polarizing beam splitter to form a ‘spin–orbit' controlled-NOT (CNOT) gate^37^. By making polarization measurements on the output spatial modes, Alice effectively made measurements in the following basis (which is mutually unbiased to the basis in which Charles applied the phases):


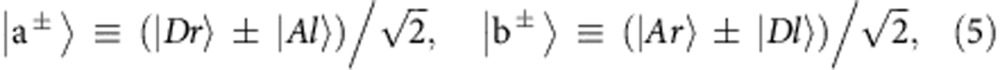


where *D* (*A*) is diagonal (anti-diagonal) polarization. The two-photon four-qubit state can then be written as:


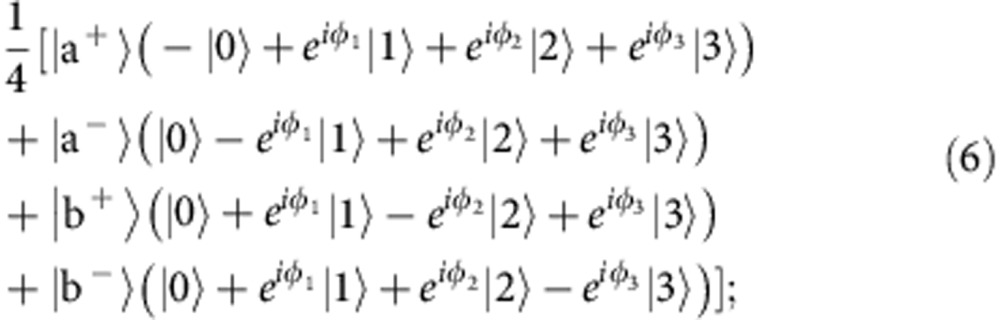


here, states |a^±^〉 and |b^±^〉 refer to Alice's photon, while |0〉, |1〉, |2〉 and |3〉 refer to Bob's. Therefore, Alice's measurement projects Bob's photon into a state that can be corrected by making a *π*-phase shift on the relevant term.

### State verification through tomographic reconstruction

In our proof-of-principle experiment, we did not apply these phases for each photon as its partner was detected, which would have required photon storage and feed-forward state correction (see Supplementary Note 1). Instead, Bob performed a full two-qubit single-photon tomography on his photon using liquid crystals and a scanning hologram^33^. Bob's hologram, like Charles', was used in conjunction with single-mode fibres to convert a particular spatial mode into a Gaussian mode in the ±1 diffraction orders; using this technique, it was possible to make spatial-mode measurements on the photons in different bases. Since different regions of the hologram (used in conjunction with single-mode fibres) converted different spatial modes to Gaussians, taking a complete polarization tomography at each hologram region enabled a tomographically overcomplete set of polarization and spatial-mode measurements on Bob's photon ({*H*, *V*, *D*, *A*, *R*, *L*})⊗({*h*, *v*, *d*, *a*, *r*, *l*}), where (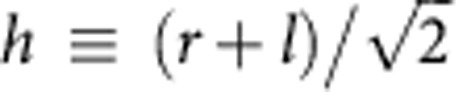, 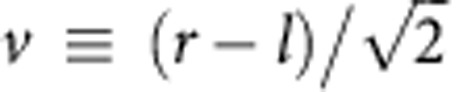, 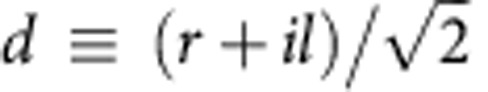, and 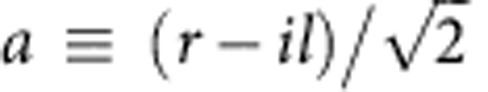) (ref. 33). Correlating Bob's measurement outcomes with Alice's, we used maximum likelihood state reconstruction^38^ to determine what state *ρ* Bob received for each of Alice's measurement outcomes. Finally, we then numerically applied the transformation indicated by Alice's measurement outcome to the reconstructed states, to compare with the original state intended to be transmitted.

**Figure 3:**
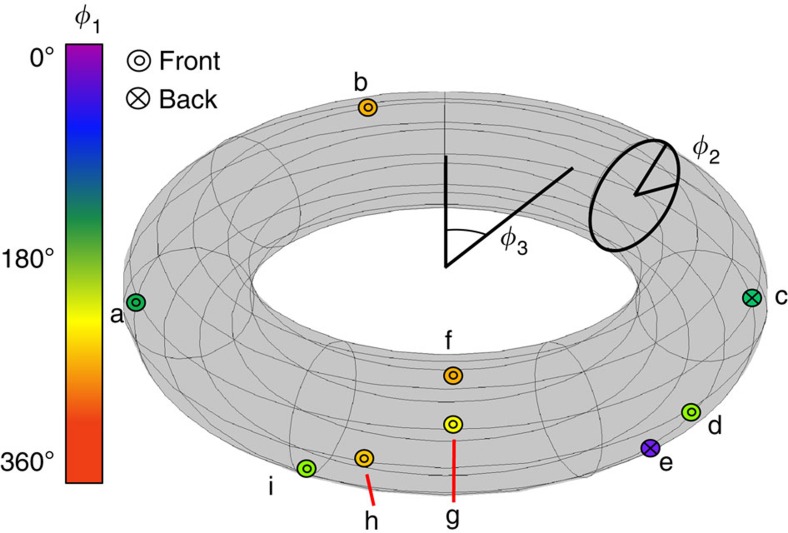
A visual representation of the distributions of the states communicated from Alice to Bob using SDT. The states that were measured can be represented as lying on a three-dimensional hyper-torus (one dimension for each state parameter) embedded in a six-dimensional Euclidean space for general ququart states. The average fidelity of all teleported states was 87.0(1)%. ‘Front' in the legend refers to point locations on the side towards the viewer in this perspective, while ‘back' refers to those obscured by the front surface. The *φ*_1_ parameter can be read from the fill-colour of the circle surrounding the point locator on the surface of the torus parameterized by *φ*_2_ and *φ*_3_.

**Figure 4:**
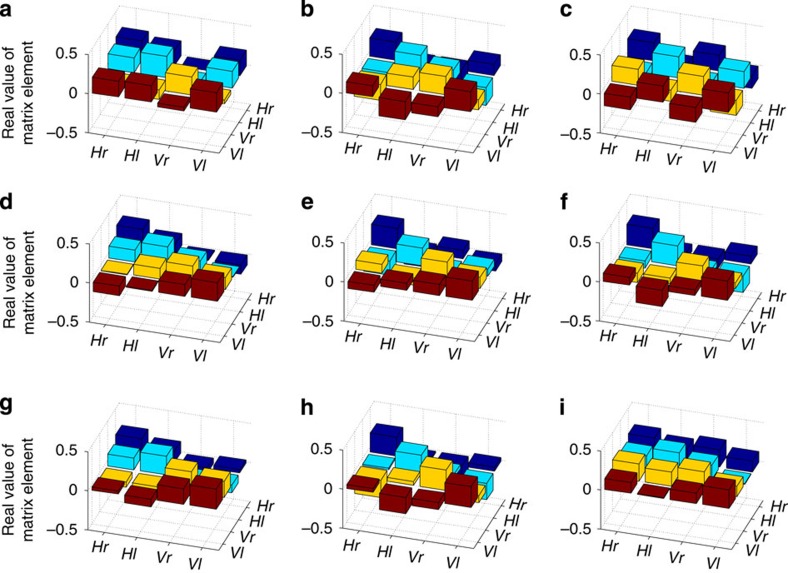
Reconstructed density matrices of transmitted states. Each of the panels above corresponds to a quantum state transmitted from Alice to Bob using superdense teleportation. For each of these states, Bob performed a full two-qubit quantum state tomography in polarization and orbital angular momentum. By correlating his measurements with Alice's measurement results and numerically performing corrective transformations, Bob reconstructed the density matrix of the state that Alice transmitted. Each panel shows the reconstructed density matrices (for clarity we display only the real parts of these matrices) in the horizontal and vertical polarization basis (H and V) and the right and left orbital angular momentum basis (r and l). The panel labels (**a**–**i**) correspond to the letters in Fig. 3 and Table 2, which list the target states and state fidelities.

The average fidelity over all the measured teleported states (see Figs 3 and 4) was 87.0(1)%, approximately twice the 44% average fidelity limit for sending a single equimodular ququart state over a classical channel without entanglement (see Methods). For comparison, perfect QT of a qubit exceeds the classical limit by 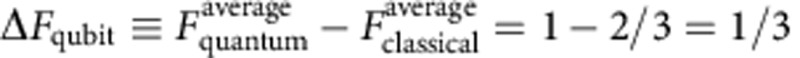 (ref. 39), and actual achieved results are lower, often much lower. In addition, recent improvements in spatial-mode sorting could increase the fidelity of SDT even further^40^.

**Figure 5:**
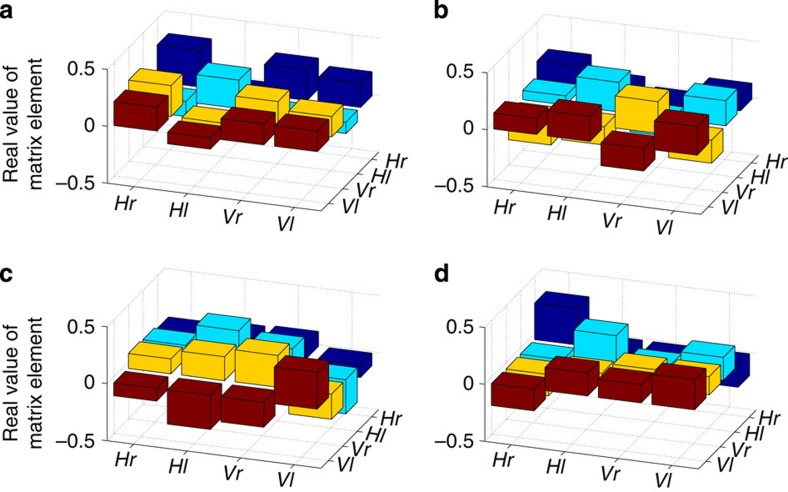
Reconstructed density matrices without Bob's corrections. Each of the four panels shown above corresponds to the state that Bob receives for each of Alice's four measurement outcomes. For each state that Charles prepared, Alice measured in a basis that is mutually unbiased in the basis in which Charles applied phases. Each of Alice's measurement outcomes projected Bob's photon into one of four orthogonal states. By performing a two-qubit polarization and orbital angular momentum tomography in coincidence with Alice's measurement, Bob reconstructed the density matrix of each of these states. (**a**–**d**) Each of these four density matrices (for clarity we display only the real parts of these matrices) are in the horizontal and vertical polarization basis (H and V) and the right and left orbital angular momentum basis (r and l). The major limiting factor of fidelity in these reconstructions is the crosstalk between orbital angular momentum modes indicated by the differing values of the diagonal elements. After these matrices were reconstructed, Bob numerically performed the corrective transformations to obtain the final reconstructed states shown in Fig. 4.

As seen in Fig. 5, the diagonal elements of our reconstructed density matrices are not all equal, in contrast to the theoretical expectation for equimodular states (see Methods). This inequality appears to arise from spatial-mode crosstalk in both Alice's and Bob's measurements; such crosstalk is the main limitation in the fidelity of the reconstructed states. We also examined how well each of the phases that Charles sent was transferred from Alice to Bob. From our state reconstructions, we estimate that the systematic error in the phases of Bob's reconstructed states was ±4.0° for each *φ*_a_, *φ*_b_ and *φ*_c_ (see Fig. 2 for definitions of these phases in terms of *φ*_1_, *φ*_2_ and *φ*_3_). This deviation suggests that Charles and Alice can reliably communicate nearly 10^5^ 
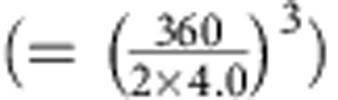
 distinguishable states to Bob.

**Figure 6:**
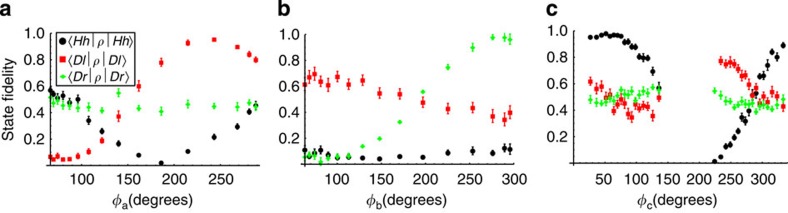
Measurement fringes as a function of Charles' phases. For each panel only one phase is varied while the others are fixed. (**a**) The theoretical fidelity range (Δ*f*≡maximum−minimum) for the black, red and green curves are Δ*f*=0.5, 1 and 0, respectively; (**b**) the theoretical fidelity range values are Δ*f*=0, 0 and 1, respectively; (**c**) theoretical fidelity range values are Δ*f*=1, 0 and 0, respectively. Error bars represent s.d. and are calculated through Monte Carlo simulation assuming Poissonian statistics. The larger-than-predicted fidelity range of some of these curves arises from crosstalk in the polarization/spatial-mode measurements. The missing strip of data around 180° in the final figure was due to instability in the active feedback system used to stabilize our interferometer at angles near this value of *φ*_c_.

In addition to the full state tomographies, we also made partial reconstructions over a much larger number of input phases, to verify that Charles and Alice could teleport a wide range of phase settings to Bob. For these measurements, Charles varied one of the three phases while keeping the others constant. Then, instead of making all 36 measurement configurations for a full two-qubit tomography for each of Charles's phase settings, Bob only made specific measurements to find the values of the three interferometric functions, which varied with phase (〈*Hh|ρ|Hh*〉, 〈*Dr|ρ|Dr*〉 and 〈*Dl|ρ|Dl*〉, where *ρ* is the state of Bob's photon). Each of these measurements varied uniquely with each of the phases Charles applied, resulting in phase-dependent fringe curves (see Fig. 6). Some measurements displayed unexpected phase dependence, varying with phases of which they were supposedly theoretically independent. This deviation from the expected measurement/phase relationship is further evidence that spatial-mode crosstalk is a limiting factor in this proof-of-principle experiment.

## Discussion

All improvements in both classical communication cost and experimental simplification can be associated with the shape of the constrained space in which the equimodular states reside, specifically, a type of hyper-torus, which is topologically different from the space associated with general quantum states of the same number of parameters. Because of this topological difference, it is possible to perform universal (within the restricted portion of the space) NOT gates, which are impossible to implement for general quantum states^15^. As was previously mentioned for qubit RSP, it is the impossibility of this operation over general quantum states that requires Alice to use a POVM and two classical bits in RSP to send Bob enough information to transform his state to the target state. In one-parameter SDT, the ‘universal' NOT gate (mapping all one-dimensional equimodular states to their orthogonal) required for Bob to recover the target state is just a simple π-phase shift between two basis states. When moving to higher-dimensional spaces, Bob must be able to perform an entire set of universal NOT gates that transform an input state to each of its orthogonal states. Again, these transformations are impossible to implement for general qudit states, but are simple relative phase shifts for inputs restricted to the set of equimodular states used in SDT.

The topological structure of equimodular states also influences their information content. In particular, the parameters of an equimodular state sweep out a more significant portion of Hilbert space than an equivalent number of parameters in a lower-dimensional general quantum state (for example, 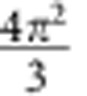 versus 
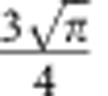
 for two-parameter state communication using SDT or QT using *n*=3 in Supplementary equation 1 and *m*=2 in Supplementary equation 2, respectively) (see Methods and Supplementary Notes 2 and 4). In fact, the ratio of the volume of the space of equimodular states to the corresponding space of general quantum states grows exponentially with the number of state parameters (see Methods). This volume ratio is related to the ratio of the number of states that can be ‘packed' into the two volumes: as the dimension increases, an exponentially larger number of statistically distinguishable states (for a small minimum statistical distance between states) can be packed into the class of equimodular states (hyper-torus) than into the class of general states (hypersphere) with the same number of state parameters. A second perspective of why equimodular states have greater information content can be understood by examining the amount of information that can be inferred about general and equimodular states from a single-shot measurement. We define classical teleportation as the optimal strategy for guessing a quantum state given a single-shot measurement^39^, which is equivalent to the optimal strategy for Alice to communicate an unknown state to Bob by sending the result of a single measurement without shared entanglement. With this definition, the average fidelity of classical teleportation is lower for the equimodular states used in SDT 
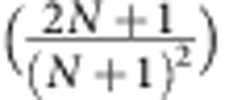
 than for general quantum states 
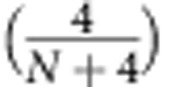
 of the same number of quantum parameters (*N*) used in QT and RSP (see Methods)^41^. This is an indication that SDT not only requires transmitting fewer classical bits to teleport the quantum parameters, but that these parameters in some sense contain more information on average than the parameters of general states used in RSP and QT.

We have implemented an entanglement-enhanced quantum state communication protocol that can communicate quantum state parameters with less classical information transfer and simpler measurements than standard QT or RSP. Using SDT we were able to transfer a wide variety of states from Alice to Bob with much better fidelity than classical teleportation. In addition to the pure target states that were teleported in this experiment, these techniques might also be extended to transfer partially mixed equimodular states as well. We also speculate that SDT might be used to exchange quantum state inputs between a client and quantum server in blind quantum computing^42^. It should be noted that both SDT and RSP are closely related to quantum steering^43^. We are currently investigating the application of recent advances in quantum steering and semidefinite programming to the quantum states reconstructed in this experiment^44^,^45^. Because universal NOT operations can be performed on equimodular states, they might also have interesting applications in ideal quantum cloning^46^ and in dynamical decoupling noise-reduction techniques^47^. Finally, this research shows that equimodular states have topological features that might make them superior to general states for quantum state communication (that is, the power of SDT comes from the fact that equimodular states are topologically different from general quantum states) and motivates further investigations into how such constrained states might be used to optimize other quantum information techniques. For example, equimodular states are precisely those necessary to implement the quantum ‘fingerprinting'^48^.

## Methods

### Source details

The source of entangled photons used in our experiment was created by pumping two orthogonally oriented 0.6-mm-thick BBO crystals with a 351-nm Ar^+^ laser focused to a 90-μm beam waist. The optic axis of the BBO crystals was oriented such that the 702-nm downconversion photons exited the crystal with a half-opening angle of 3°. The downconversion was then filtered by 10-nm full-width at half-maximum interference filters and measured using PerkinElmer single-photon counting modules with a coincidence window of 10 ns. For states b, f, g and e (as referenced in Fig. 3 in the main text) hyperentangled photons were generated and measured with a total coincidence rate of ∼90 s^−1^ into all detected single modes, with a ∼0.6% heralding efficiency of the teleported state. For states a, c, d, h and i, a different pump power was used resulting in a measured coincidence rate of ∼140 s^−1^ and a heralding efficiency of ∼0.5%. The majority of loss in the system arises from transmission through holograms and liquid crystals, imperfect coupling into single-mode fibres and imperfect detection by the single-photon counting modules.

### State reconstruction

A full two-qubit polarization and spatial-mode tomographic reconstruction was performed for each state that was transmitted from Alice to Bob using superdense teleportation. The density matrices representing these states were then calculated using maximum likelihood state estimation techniques^38^. However, each of Alice's detectors heralds a different state on Bob's side, so a simple reconstruction on Bob's photon without accounting for Alice's measurement yields a mixed state. To reconstruct the state that Bob would have measured had he made the corrective unitary transformation on his photon based on Alice's message, state tomographies were performed in coincidence with Alice's measurements (see Fig. 5; the four matrices (one for each of Alice's measurements) that were then averaged after numerically applying the respective unitary transformations (see Fig. 4)). The phase angles (corresponding to the parameters that Charles encoded) and the fidelity with the target state were calculated for each of the resulting density matrices (see Table 2).

### Packing number and volume ratio

One way to measure the complexity of a set of states is to consider the ‘packing number' with respect to the Bures distance^49^. For states with an angle less than 
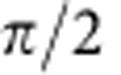
, the Bures distance coincides with the usual Euclidean distance, which will be used here for simplicity.

Given a subset *T* in a *d*-dimensional real Euclidean space **R**^*d*^, we define the packing number *P*(*T*, *δ*)=max *N* as the maximal number of points *x*_1_, …, *x*_*N*_ in *T* such that 
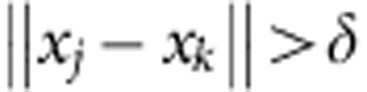
 for all 1≤*j*≠*k≤N*. If *T* is sufficiently smooth and of dimension *n*, we have:


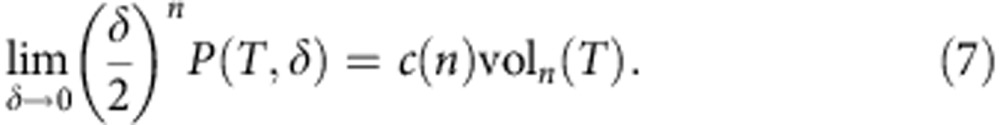


Here vol_*n*_(*T*) is the *n*-dimensional measure^50^ and *c*(*n*) is the packing density of Euclidean space. It is known^51^ that 
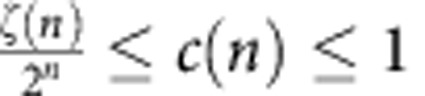
 holds for the Riemann *ζ*-function. The estimate *c*(1)=1 is easy, and 
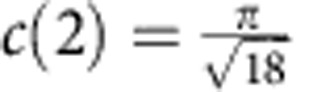
 is due to Gauss. For larger dimension, only lower and upper estimates are known, see for example, ref. 52.

For the set of equimodular states *T*_*n*_=*n*^−1/2^**T**^*n*^⊂**C**^*n*^, *d*=*n*−1 (because **C**^*n*^=**R**^2*n*^ and there are *n*−1 real parameters in *T*_*n*_). Using the *d*=*n*−1 Haussdorff measure on the scaling factor of *T*_*n*_, we find


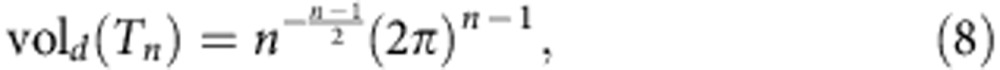


where we divide by 2*π* to account for the fact that a global phase does not change the state. Let us compare this with a sphere of dimension *d*, i.e., the set of vectors *S*^*d*^⊂**R**^*d*+1^ of points of length 1. Let us denote by *B*_*d*+1_ the unit ball in **R**^*d*+1^, that is, the set of all points of length less than one. It is well known that


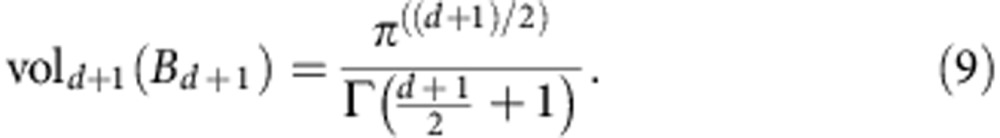


Using Stirling's formula 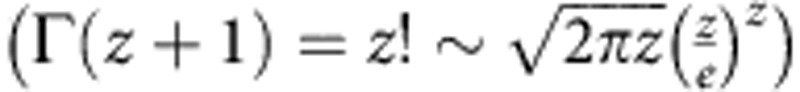 we arrive at 


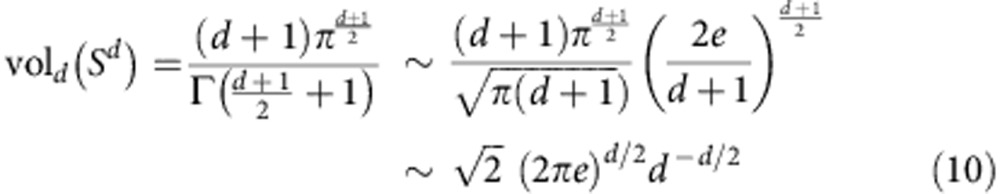


Here the ∼ symbol denotes that if *a*_*d*_∼*b*_*d*_ then 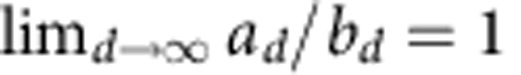. The error of this approximation can be reduced by including higher-order terms when approximating the Γ-function. For *d*=*n*−1 we find


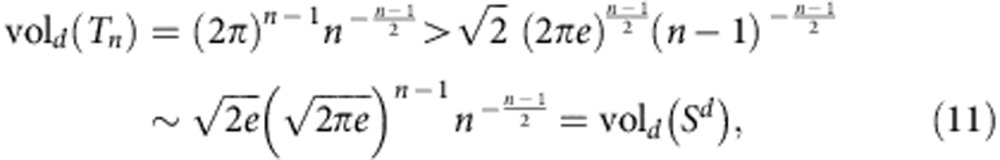


because 
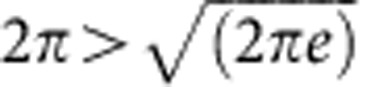
. Thus, the packing number for a torus is larger than the corresponding packing number for a sphere of the same dimension, assuming small *δ*. For these calculations, we have assumed that the general class of states is embedded in a sphere instead of a complex projective space. However, a full calculation in complex projective space still shows that the ratio of the volumes of equimodular versus general states of the same number of parameters grows exponentially (see Supplementary Note 2). In realistic quantum communication experiments, systematic error and shot noise will reduce the distinguishability of neighboring quantum states. This reduction will put a lower bound on the minimum statistical distance (*δ*) by which two states can be separated and still be experimentally distinguished. To address this issue, we have also considered the packing number for equimodular versus general quantum states for a fixed threshold *δ* (see Supplementary Note 3).

### Classical teleportation fidelity

To establish the optimal average fidelity with which a *d*-dimensional quantum state can be transmitted over a classical channel without entanglement, we must determine how well the state can be estimated with a single optimal measurement. Alice then makes this measurement and sends the result to Bob (who knows Alice's measurement strategy), who makes a state estimation based on this message. The best average fidelity that one can achieve using this classical teleportation strategy has been calculated to be^41^:


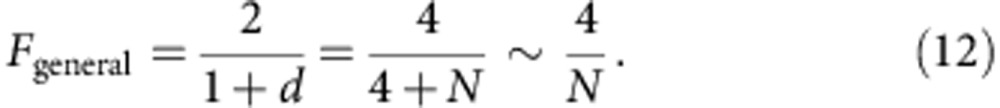


Because the hyper area of an equimodular state is larger than that for a general quantum state of the same number of parameters, the former are more difficult to send classically. The average fidelity of an optimal state estimation strategy can be calculated using well-established methods of Massar and Popescu^39^. It is optimal to measure in a basis that is mutually unbiased to the basis in which Charles applies the phases, for example:


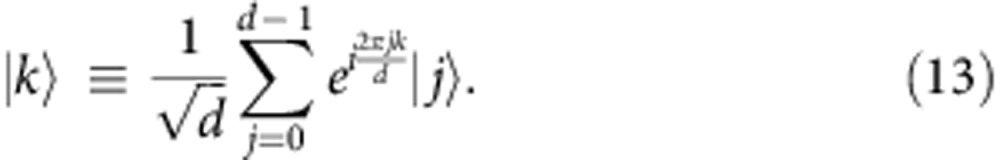


If Alice measures in this basis and sends the result to Bob, it is the optimal strategy for Bob to simply guess the same state that Alice measured. The average fidelity of his state is then:


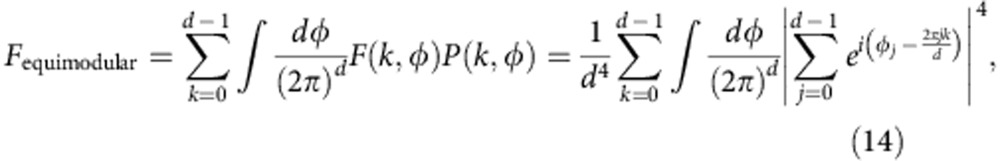


where *P*(*k*, *φ*) is the probability that Alice detects state *k* of the measurement basis, *F*(*k*, *φ*) is the fidelity of Bob's guess, given that Alice measured state *k*, and *φ* is the set of phases that parameterize the set of equimodular states. After shifting phase angles by 
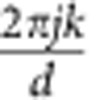
 (by a simple redefinition), we simplify the fidelity to:


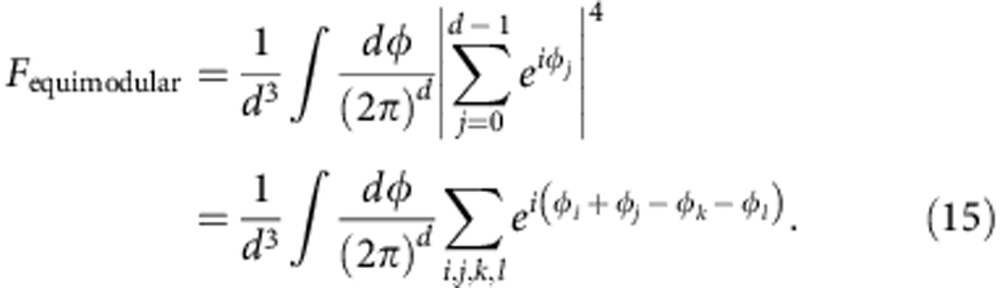


The integral is unity for all phase combinations that add to zero, and zero for all other combinations:


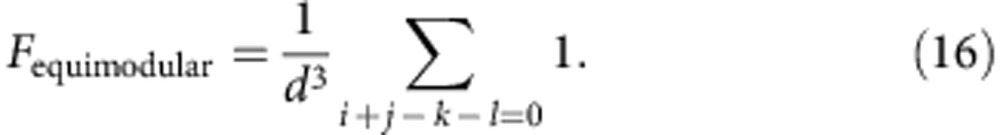


These indexes add to zero when *i=k* and *j=l* (occurs *d*^2^ times) and when *i=l* and *j=k* (occurs *d*^2^ times); however, this double-counts when *i=k=j=l* (occurs *d* times). Therefore, the optimal average fidelity for an equimodular state becomes:


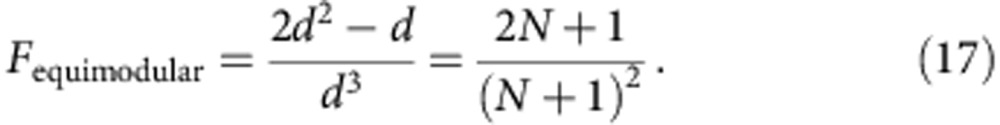


Thus, we see that the average classical teleportation fidelity for equimodular ququart states is 44%. Examining the asymptotic behaviour of the average fidelity for large *N* 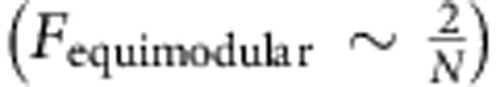 of general states (equation (12)) versus equimodular states (equation (17)), we see that equimodular states can be transmitted over a classical channel (without entanglement) with on average half the fidelity that general states (with the same number of state parameters) can be transmitted.

## Additional information

**How to cite this article:** Graham, T.M. *et al*. Superdense teleportation using hyperentangled photons. *Nat. Commun.* 6:7185 doi: 10.1038/ncomms8185 (2015).

## Supplementary Material

Supplementary Information Supplementary Figures 1-2, Supplementary Notes 1-4 and Supplementary References

## Figures and Tables

**Figure 1:**
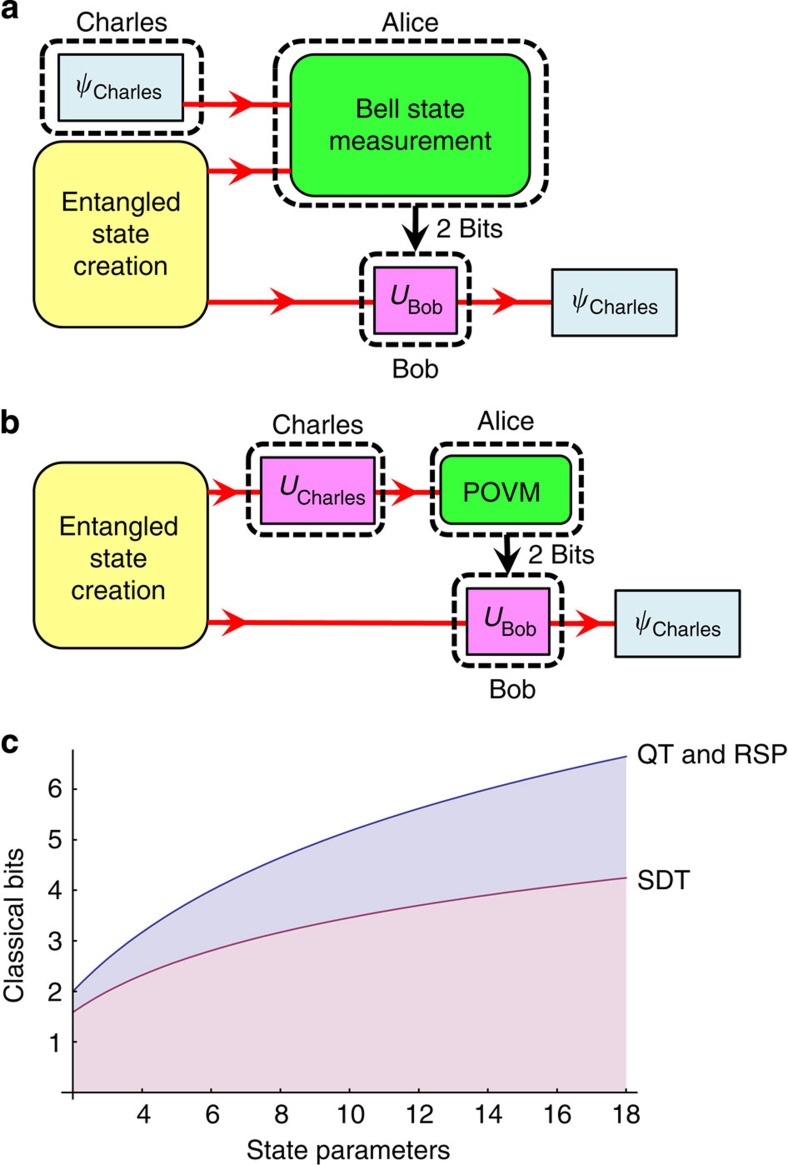
Schemes to transfer one qubit. (**a**) Quantum teleportation layout. Charles prepares a state for Alice, who performs a Bell measurement between her state and Charles'. She then transmits the outcome to Bob, who is able to transform his photon into the state Charles had chosen. (**b**) Remote state preparation (RSP) layout. Charles performs a unitary transformation on one photon and sends it to Alice, who makes a positive-operator valued measure (POVM) measurement on the state. She then sends the outcome to Bob, who transforms his state into the state Charles chose. (**c**) The required number of transmitted classical bits for quantum teleportation (QT), RSP and superdense teleportation (SDT) as a function of the number of parameters teleported; for a large number of parameters, the ratio of classical bits needed for QT and RSP to bits needed for SDT approaches 2.

**Table 1:** Resources required to send *N* state parameters with 100% fidelity for each technique using linear optics.

	**State dimension**	**Success probability**	**Classical bits**	**Alice detector #**	**Bob transformation #**	**State known to Charles**
QT	2	1/2	2	4	4	Optional
RSP (probabilistic)	(*N*+2)/2	2/(*N*+2)	1	1	1	Required
RSP (deterministic)	(*N*+2)/2	1	2Log_2_[(*N*+2)/2]	[(*N*+2)/2]^2^	[(*N*+2)/2]^2^	Required
SDT	*N*+1	1	Log_2_[*N*+1]	*N*+1	*N*+1	Required

Superdense teleportation (SDT) uses a high-dimensional input state to transmit quantum state parameters with less classical communication and fewer detectors and transformations than both standard quantum teleportation (QT) and deterministic remote state preparation (RSP). While probabilistic RSP can transmit state information with very few experimental resources, the probability of successful state transmission is inversely proportional to the number of parameters being transmitted. Note that QT of dimension *d*>2 requires nonlinear optics or the addition of *d*-entangled ancilla qubits^53^. QT is also the only technique that does not require the transmitted state to be known to a state chooser (Charles).

**Table 2:** Summary of experimental results.

**Panel of Fig. 4**	**Target phases (°)**	**Measured phases (°)**	**Average fidelity (%) with target state**
a	112, 180, 278	109.7(5), 176.0(6), 283.7(5)	86.2(3)
b	270, 90, 324	266.4(6), 80.7(7), 309.5(7)	85.7(3)
c	112, 277, 119	113.0(5), 272.4(6), 122.9(6)	87.8(3)
d	180, 180, 137	175.6(5), 176.8(5), 141.2(6)	86.9(3)
e	26, 202, 145	23.7(4), 204.4(5), 154.9(4)	86.4(2)
f	270, 90, 184	262.0(7), 80.8(6), 193.7(7)	86.8(3)
g	211, 158, 185	208.5(4), 162.9(4), 191.0(4)	88.4(3)
h	268, 148, 209	273.2(5), 141.4(5), 208.9(6)	86.2(3)
i	180, 277, 223	176.4(5), 272.6(5), 222.6(6)	89.2(2)

Bob's measurement results for each set of target phases chosen by Charles. For every teleported state Bob received, a density matrix was reconstructed that was used to calculate the measured phases and the fidelity of teleported state with the target state.
