# Supplementary material for: Superdense teleportation using hyperentangled photons
Source: Nat Commun. 2015 May 28;6:7185. doi: 10.1038/ncomms8185 (PMC4458874; doi:10.1038/ncomms8185)
Supplement: Supplementary Information — Supplementary Figures 1-2, Supplementary Notes 1-4 and Supplementary References [file ncomms8185-s1.pdf]

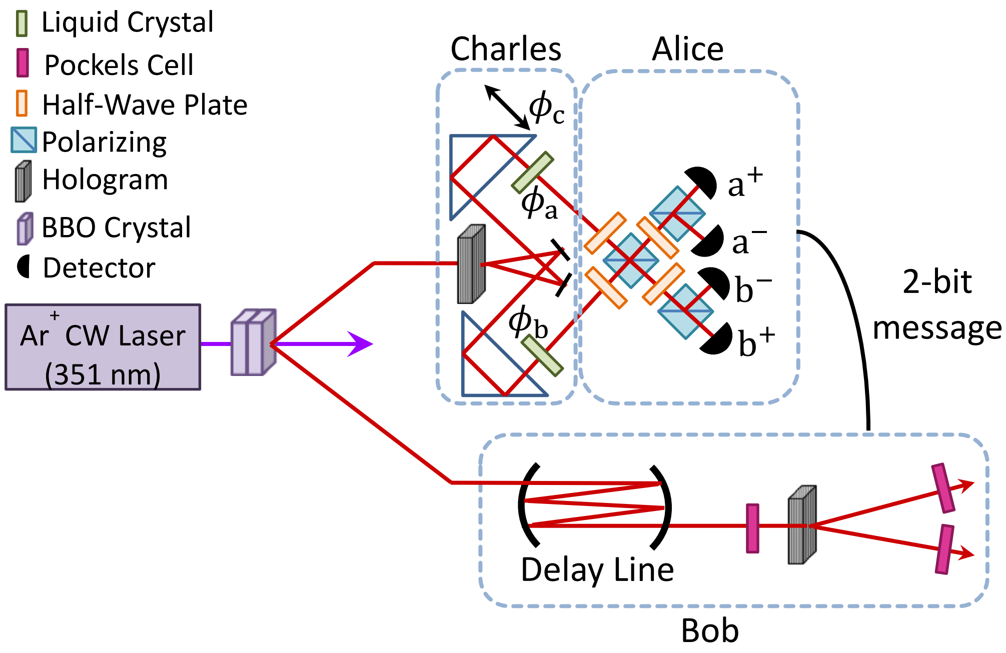

Supplementary Figure 1 | A possible layout for implementing SuperDense Teleportation with feed-forward correction. The addition of a delay line and Pockels cells allows Bob to store his photon until he receives the result of Alice's measurement and quickly make the corrective unitary transformation based on her message.

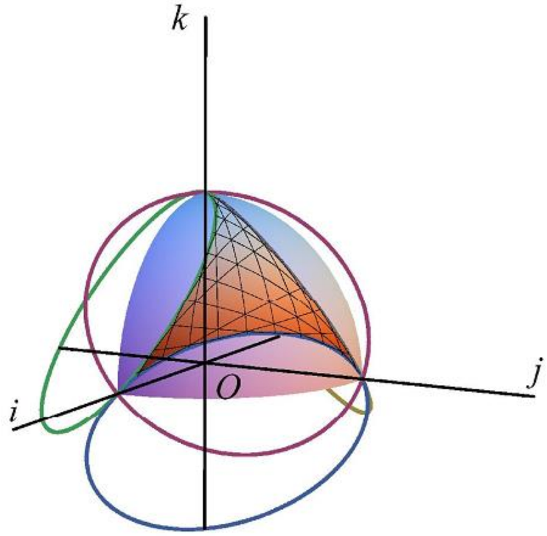

Supplementary Figure 2 | A visual representation of measurement outcomes on equimodular states. The orange triangular shaded area is the set of points giving the measurement outcome probabilities accessible by measuring equimodular qutrits in a basis mutually unbiased to the basis in which the two phases are applied. This area covers part of the positive octant on the unit sphere (the blue-purple-pink region) where vector *i*, *j*, and *k* correspond to the three orthogonal measurement outputs, where probabilities must be normalized (i.e., $i^2 + j^2 + k^2 = 1$). The colored circles geometrically define the shaded region. The blue, green, and yellow circles are pairwise tangent to each other at the points (1,0,0), (0,1,0), and (0,0,1), defining a fourth circle (shown in purple). These form a one-parameter subset of all accessible outputs which define the boarder of the shaded region. By plotting only the absolute value of these amplitudes, we find the part of the octant inside the shading. This region can be geometrically shown to encompass ~31% of all possible count rate combinations (i.e., 31% of the octant *i*, *j*, and *k*>0), using the circles shown on this diagram.

Supplementary Note 1: Feed-Forward Corrections

While we did not perform feed-forward state correction in this experiment, we can outline one possible way to extend our implementation to allow Bob to perform corrective transformations on his photons based on Alice's measurement outcomes. For this extension, two main modifications must be made to our implementation (see Supplementary Figure 1). First, Bob must have a way of storing his photon to allow Alice time to measure her photon and transmit the outcome to Bob. Perhaps the easiest way to implement such a delay is to use an optical delay line. Secondly, Bob must possess a way of quick applying the corrective unitary transformations in the path of his photon allowing him to convert his photon to the target state based on Alice's message. Bob could make these transformations quickly using Pockels cells placed before and after his hologram and thus perform full feed-forward correction of his state.

Supplementary Note 2: Volume Estimates

The estimates in the Methods section used the simplified assumption that the set of states is embedded in a sphere. More correctly, we should perform the same calculations for the projective space $CP_m = S^{2m-1}/\mathrm{T}$ of rank-one density, where CP_m denotes an m-dimensional complex projective space (corresponding to an m-dimensional general quantum state), S^{2m-1} represents a (*2m-1*)-dimensional sphere, and T is the compact unitary group (also known as $U(1)$). In this equation, we identify vectors with the same global phase factor. We now show that using projective spaces leads to similar estimates as calculated in the manuscript. A key point is that if a space can be identified as the quotient of unimodular groups, then via the Haar measure (the respective groups' invariant distance measure), its volume is the ratio of the volumes of the corresponding groups.

The volume calculation for equimodular states can be readily computed from the definition of these states. Equimodular states of dimension n are defined by points on $T_n = n^{-1/2}\mathrm{T}^n \subset \mathbb{C}^n$, up to a global phase factor, where points in T^n are labeled by $(e^{i\phi_1}, e^{i\phi_2}, \cdots, e^{i\phi_n})$. Integrating over these phases and dividing by T_1 to account for the fact that an overall phase factor does not change the state, we see that the volume of equimodular states is given by

$$\mathrm{vol}_{n-1}\left(T_n / T_1\right) = \left(2\pi\right)^{n-1} n^{-(n-1)/2}, \tag{1}$$

where the $n-1$ subscript refers to the dimension of the space that the volume is calculated in.

To compute the volume of general states, we first need to find the Haar measure on the surface of a $2m$-dimensional sphere (i.e., $S^{2m-1} \subset \mathbb{R}^{2m}$). This can be obtained from identifying S^{2m-1} as a quotient of two orthogonal groups O_{2m}/O_{2m-1}. As multiplication by a phase factor in S^{2m-1} is again a group action of the compact group T, the complex projective space can be identified as a quotient of groups $CP_m = O_{2m}/\mathrm{T}\times O_{2m-1}$. Therefore [1, 2], we deduce that the volume of the set of m-dimensional general states is

$$\begin{aligned}
\mathrm{vol}_{2m-2}\left(CP_m\right) &= \frac{\mathrm{vol}_{2m-1}\left(S^{2m-1}\right)}{2\pi} = \frac{\left(2m-1\right)\dfrac{\pi^{(2m-1)/2}}{\Gamma\left(\dfrac{2m-1+1}{2}+1\right)}}{2\pi} \\
&= \frac{\left(2m-1\right)}{2}\frac{\pi^{(2m-3)/2}}{m!} \\
&\sim \frac{\left(2m-1\right)\pi^{(2m-3)/2}}{2\sqrt{2\pi m}} e^m m^{-m} \\
&\sim \frac{1}{\sqrt{2\pi^2}} m^{-1/2}\left(\pi e\right)^m m^{-m},
\end{aligned} \tag{2}$$

where Γ denotes the gamma function and Stirling's approximation was used to simplify the factorial.

We can now compare volumes for equimodular (Eqn. 1) and general states (Eqn. 2) with the same number of state parameters $N \equiv 2(m-1) = n-1$. Thus, for *n* odd we use $m = 1 + \frac{n-1}{2}$ and find the volume for general states is

$$\begin{aligned} \mathrm{vol}_{2m-2}(CP_m) &\sim \frac{\pi e}{\sqrt{2}\pi^2}\left(1+\frac{n-1}{2}\right)^{-1/2} (\pi e)^{(n-1)/2}\left(1+\frac{n-1}{2}\right)^{-\left(1+\frac{n-1}{2}\right)} \\ &\sim \frac{\pi e}{\sqrt{2}\pi^2}\left(\frac{n+1}{2}\right)^{-3/2} (2\pi e)^{(n-1)/2}\, n^{-(n-1)/2}. \end{aligned} \tag{3}$$

Comparing the volume of the set of equimodular states (Eqn. 1) to the volume of the set of general states with the same number of state parameters (Eqn. 3), we find the ratio of the two volumes is

$$\begin{aligned} \frac{\mathrm{vol}_{n-1}(T_n / T_1)}{\mathrm{vol}_{n-1}\left(CP_{1+\frac{n-1}{2}}\right)} &= \frac{(2\pi)^{n-1} n^{-(n-1)/2}}{\frac{\pi e}{\sqrt{2}\pi^2}\left(\frac{n+1}{2}\right)^{-3/2} (2\pi e)^{(n-1)/2} n^{-(n-1)/2}} \\ &\sim \frac{(2\pi)^{n-1}}{(\sqrt{2\pi e})^{n-1}} \end{aligned} \tag{4}$$

where again we observe that $2\pi > \sqrt{2\pi e}$ implies that the volume of the equimodular states in projective space is larger than the volume of general state space with the same number of parameters. Let us note in passing that the volumes for the sphere and the projective space have the same leading term for large *n*.

As a conclusion, we see that, confirming the calculation in the manuscript, the equimodular states occupy a larger volume, and hence more of these states can be statistically distinguished for small δ, the minimum distance between the packed states. Some of this advantage, however, is obscured by the difficulty of finding the optimal packing configuration and the unknown density of this packing configuration. Indeed, the volumes differ by at most a factor 2^n, and hence, in practice, we would need very precise configurations to realize this advantage. Finding optimal packing configurations is a mathematically hard problem which remains unsolved despite centuries of research.

Supplementary Note 3: General Packing Numbers and Distinguishability of Equimodular versus General States

The preceding volume approach determines packing numbers for infinitesimal values of δ, and the rate of convergence may depend heavily on the *shape* of the manifold. Experimentally, limited numbers of state copies as well as systematic noise will increase δ. We will now show that for an equal number of state parameters, there are $2^{c_1 n}$ general states and $2^{c_2 n}$ equimodular states separated by a minimum distance δ, where c_1, c_2, and δ are constants which are independent of the state dimension. For technical reasons it is better to work with

the so-called entropy numbers, defined as follows: for some set $K \subset \mathbb{R}^d$ the *entropy number* of that set is $N(K,\varepsilon) = \min k$, where there are points $x_1,\ldots,x_k \in \mathbb{R}^d$ with

$$K \subset \bigcup_i x_i + \varepsilon B_d \,. \tag{5}$$

Here $B_d = \{x \mid \|x\| \le 1\} \subset \mathbb{R}^d$ is the d-dimensional unit ball. Intuitively, entropy numbers are the minimal number of overlapping ε-radius balls to cover the set K. In contrast, the packing number, $P(K,\varepsilon)$, is intuitively the *maximal* number of $\varepsilon/2$-radius balls (thus the distance between any two balls is at least ε) that can be squeezed inside K. It is thus easy to see that $P(K,2\varepsilon) \le N(K,\varepsilon)$. The "pigeonhole principle" [3] implies $N(K,\varepsilon) \le P(K,\varepsilon)$. Thus, it suffices to study entropy numbers to estimate the scaling of packing numbers. It turns out that a lower bound on the entropy numbers can be obtained via the so-called Sudakov's inequality [4], which when applied to $T_n = n^{-1/2}\mathrm{T}^n \subset \mathbb{C}^n = \mathbb{R}^{2n}$, yields

$$\sqrt{\frac{2n}{\pi}} \le C\int_0^{\mathrm{diam}(K)} \sqrt{\log_2 N(K,\varepsilon)}\,d\varepsilon\,, \tag{6}$$

for some unknown constant C which depends on the geometry of the complex projective space.

We will now estimate the integral in eqn. (6) using the following volume estimate [4]

$$N(B_n,\varepsilon) \le \left(1+\frac{2}{\varepsilon}\right)^n . \tag{7}$$

Using logarithmic identities and setting $K \subset B_d$ we have

$$\sqrt{\log_2 N(K,\varepsilon)} \le \sqrt{\frac{2d}{\ln 2}}\sqrt{\frac{1}{\varepsilon}}\,. \tag{8}$$

To match the form of Sudakov's inequality in equation 6, we integrate equation 8 over epsilon. Breaking the integral into two intervals of [0, δ] and [δ , D] (where D is the diameter of set K) and setting $d = 2n$ and $D \le 2$, we have

$$\begin{aligned} C\int_0^{\mathrm{diam}(K)} \sqrt{\log N(K,\varepsilon)}\,d\varepsilon &= C\int_0^{\delta} \sqrt{\log N(K,\varepsilon)}\,d\varepsilon + C\int_{\delta}^{D} \sqrt{\log N(K,\varepsilon)}\,d\varepsilon \\ &\le \sqrt{\frac{4n}{\ln 2}}\,C\int_0^{\delta}\sqrt{\frac{1}{\varepsilon}}\,d\varepsilon + (2-\delta)C\sqrt{\log N(K,\delta)} \\ &\le C\sqrt{\frac{4n}{\ln 2}}\,2\delta^{1/2} + 2C\sqrt{\log_2 N(K,\delta)}\,. \end{aligned} \tag{9}$$

Thus, we have

$$\sqrt{\frac{2n}{\pi}} \le C\sqrt{\frac{4n}{\ln 2}}\,2\delta^{1/2} + 2C\sqrt{\log_2 N(K,\delta)}\,. \tag{10}$$

It is now possible to find a δ independent of dimension n such that the inequality in equation (10) holds. For example, taking the first term on the right-hand side to be equal or smaller than

half the part of the expression on the left-hand side, $C\sqrt{\frac{4n}{\ln 2}}2\delta^{1/2} \le \frac{1}{2}\sqrt{\frac{2n}{\pi}}$, i.e. $\delta \le \frac{\ln 2}{32\pi C^2}$, we find $\sqrt{\frac{n}{8\pi C^2}} \le \sqrt{\log_2 N(K,\delta)}$. This, combined with equation (7) and the fact that $P(K,2\delta) \le N(K,\delta)$, leads to the following result:

Theorem: There exists a δ and $C > 0$ (independent of n) such that the packing number for general quantum states is bounded

$$e^{\frac{2n}{\delta}} \ge P(K,\delta) \ge 2^{\frac{n}{8\pi C^2}}. \tag{11}$$

This means that if we can statistically distinguish states separated by a distance δ, than we can encode at least $\frac{n}{8\pi C^2} \equiv c_1 n$ many bits for $\delta \le \frac{\ln 2}{32\pi C^2}$. Using similar arguments one can show that even considering restricted equimodular states with phases restricted to 0 or π, it is still possible to encode bits.

In summary, using the freedom of encoding in the whole sphere we see that only $c_1(\delta)n$ many bits can be encoded with mutual distance δ. Despite not knowing the exact behavior of packing numbers, we can determine that for some minimal statistical distance between states, there is a constant ratio of bits that can be encoded in each volume, which is independent of dimension.

Supplementary Note 4: Measurement Outcomes of Equimodular States

In the previous two sections, we examined the number of states that could be packed into the class of equimodular states compared with general quantum states with the same number of state parameters (but different state dimension). It is also instructive to compare equimodular states to general states with the *same* state dimension (and therefore double the number of state parameters). Unfortunately, these two classes of states are represented by shapes with different Euclidean dimension, since they have a different number of state parameters, making a simple volume comparison of the two shapes not very informative. A potentially more instructive comparison can be made by examining how measurements on states are affected if a state is constrained to be equimodular. The probability that a measurement on a state yields a particular result reveals information about that state. By examining how the outcome probabilities of different measurements are constrained for equimodular states, we can directly compare equimodular and general states. For example, if measurements are made in the same basis in which Charles applied his phases, then all equimodular states will give the same measurement probability signature, i.e., uniform count probability for all outcomes. This is much more constrained than general states, which can have an arbitrary normalized measurement outcome probability. However, measurements made on an equimodular state in a mutually unbiased basis will vary with the relative phase, though these measurements are still

constrained as compared to general states, and the severity of these constraints will depend on the dimension of the equimodular state. For example, just like a general qubit, measurements on an equimodular qubit state can have completely arbitrary normalized outcome probabilities. However, the ratio of possible measurement outcome probabilities of equimodular states compared to general states with the same dimension decreases as dimension increases. As an example, an equimodular qutrit (2 free parameters) accesses only ~31% of the measurement outcome probability combinations of general qutrit states (4 free parameters) (see Supplementary Figure 2). Calculating the region of outcome probabilities accessible to equimodular states becomes more difficult as the dimension of the state increases; the general calculation is beyond the scope of this work.

Supplementary References

1. Reiter, H. and Stegeman J. D., *Classical harmonic Analysis and Locally Compact Groups*, (Oxford: Clarendon Press, 1968).

2. Bengtsson, I. and Życzkowski, K., *Geometry of Quantum States: An Introduction to Quantum Entanglement*, (Cambridge University Press, 2006).

3. Dirichlet, P.G.L., *Lectures on Number Theory: Vol 16*, (American Mathematical Soc., 1999).

4. Pisier, G., *The volume of convex bodies and Banach space geometry: Vol. 94* (Cambridge University Press, 1989).
